# The impact of the COVID-19 pandemic on students’ views of a career in general practice: a focus group study

**DOI:** 10.3399/BJGPO.2023.0257

**Published:** 2024-11-13

**Authors:** Zoe Hook, Ben Jackson, Hugh Alberti, Claire Capper, Fiona Hay, Carly Hire, Hannah Randles, Juliet Zachary, Joanne Protheroe

**Affiliations:** 1 School of Medicine, Keele University, Staffordshire, UK; 2 Division of Clincal Medicine, University of Sheffield, Sheffield, UK; 3 School of Medical Education, Newcastle University, Newcastle, UK

**Keywords:** General Practice, Careers, COVID-19, Medical student

## Abstract

**Background:**

General practice is an essential part of healthcare systems in the UK and internationally but continues to struggle with recruitment. Despite this, few studies have explored factors that influence medical students’ career choices around primary care.

**Aim:**

We aimed to revisit factors that had previously been proposed following new ways of working adopted since the COVID-19 pandemic, including the impact of these changes on learning experiences in primary care.

**Design & setting:**

A qualitative study using focus groups across three English medical schools.

**Method:**

Eight focus groups were held involving 33 final and penultimate year medical students. Qualitative data were analysed using a framework approach. Transcripts were coded independently by two researchers from a different institution before themes were identified.

**Results:**

Six themes were identified: students’ prior career aspirations, their experience of the medical school curriculum, conceptualisation of general practice, future career predictions, views on the school’s curriculum philosophy, and the influence of the COVID-19 pandemic. The curriculum philosophy of each school appeared to be important in this journey and changes since the COVID-19 pandemic had an impact on all themes.

**Conclusion:**

Our study has confirmed previous findings that clinical experiences, the perceived narrative of the school, work–life balance, and working environment remain important to students in making career plans. However, in addition, we have found the changing landscape in general practice since the COVID-19 pandemic, including remote consulting, workload, continuity of care, and team-working, are additional factors that concern students.

## How this fits in

General practice is an essential part of healthcare systems but continues to struggle with recruitment. Previous factors identified affecting attitudes to general practice as a career during undergraduate study include clinical learning experiences and curriculum philosophy. Since the pandemic, remote consulting, increased workload, and isolation at work concern students when considering general practice careers. Students are apprehensive about how current challenges in general practice will affect their working lives.

## Introduction

General practice is an essential part of healthcare systems in the UK and internationally but continues to struggle with recruitment.^1,2^

Previous research has highlighted important considerations for encouraging more students to choose general practice as a career. For instance, Nicholson *et al* proposed increasing authentic exposure to placements; articulating what a ‘good GP’ is; challenging ‘discipline bashing’; and supporting positive role-modelling.^3^ In the same year, the Wass report (*By choice* — *not by chance*) identified how formal, informal, and hidden curricula affected students’ decisions about general practice, including placement time, understanding of general practice as a specialty, the negative impact of ‘GP bashing’, and the benefit of having general practice societies.^4^

In March 2020, the COVID-19 pandemic meant that authentic medical student placements were stopped, and teaching moved online. During this time general practice also changed; in July 2020 the UK Secretary of State for Health and Social Care suggested that GP consultations should be delivered remotely for the foreseeable future.^5^

This huge shift in experience may have impacted medical students’ views of general practice, with concern from students and tutors that remote consulting has limited the exposure of students to patients, especially with regard to learning examination and communication skills.^6^ Al-Bedaery *et al* found that students highly rated independent remote consultations but felt observation of remote clinics very unengaging.^7^ Students reported feeling isolated during their placements because they had no use of communal areas and because of the small amounts of time that they spent in practice, and that this changed their perception of general practice and made them less likely to pursue it as a career.^8^ However, students also report that telephone consultations allow them to learn specific skills in triaging, remote examinations, and more focused history taking.^5–7^

The denigration of general practice as a specialty choice by other medical professionals and media outlets also impacts student career choices.^9,10^ This may have been exacerbated by media criticism of general practice during the COVID-19 pandemic (in comparison to hospital doctors who were presented as ‘working hard under difficult conditions’).^11^

### Aims and objectives

Despite concerns about GP recruitment, few studies have explored factors that influence medical students’ career choices around primary care. Building on research carried out before the pandemic,^3^ we aimed to explore these factors further following the new ways of working that have been adopted since the COVID-19 pandemic, including the impact of these changes on learning experiences in primary and secondary care.

## Method

We undertook a multicentre study in three English medical schools, using qualitative methods from an interpretive paradigm. We selected focus groups as our method to encourage student dialogue and discussion and to generate ideas and theories from the students’ perspectives. The three medical schools selected varied in their size, age and time in general practice placements across their overall medical degree programmes (see Table 1).

**Table 1.** Details of the three participating medical schools

Medical school	1	2	3
Cohort size	300–400 students	180–250 students	250–350 students
Old or new^a^	Old	New	Old
Percentage of time in GP placements^b^	Below national average	Above national average	Average
Percentage of graduates entering GP training^c^	Similar to national average	Above national average	Above national average
Overview of GP placements	Several days in early years, weekly in years 3 and 4 and 7 weeks in year 5	Several days in early years, 4 weeks in years 3 and 4 and 10 weeks in year 5	Several days in early years, 6 weeks in year 3, 7 weeks in year 4
Number of focus groups held	Three	Three	Two

a Based on whether the medical school was established pre-2000 or post-2000.

b Using data from the UK National GP undergraduate survey^15^

c Using data from the last UK Foundation Programme Office career destination survey^16^

### Sampling

Researchers from each medical school invited all medical students who were in their penultimate and/or final year to participate in the study. These students would have experienced multiple clinical placements in general practice and have had experience before and during the COVID-19 pandemic. Students that were interested in participating were asked to complete a short questionnaire to understand their current career choices before participating in the focus group.

### Data collection

Eight focus groups were held across the three schools facilitated by researchers from their own institution. Two focus groups were held remotely via Microsoft Teams and six took place in-person. Written consent was obtained before or at the start of each focus group. An adaptation of the topic guide developed by Nicholson *et al* ^3^ was used, with the addition of questions around the impact of the COVID-19 pandemic. Focus group discussions were recorded and transcribed verbatim, checked and anonymised by researchers at each institution, and then shared for analysis.

### Analysis

We followed a framework analysis^12^ based on the concepts identified by Nicholson *et al* ^3^ Each transcript was coded independently by two researchers from different institutions. Researchers then met to identify, discuss, clarify, and define the main themes.

## Results

A total of 33 medical students participated across the three medical schools. Focus groups consisted of groups of two to six students; six groups were composed of final year students and two of penultimate year students.

**Figure 1.**
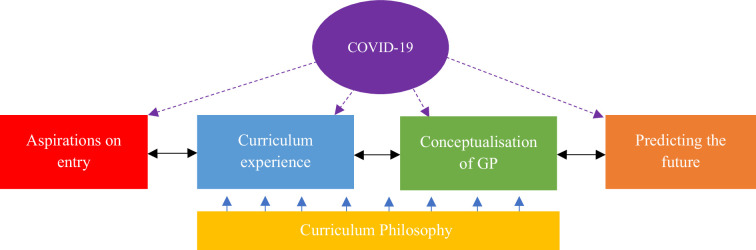
A schematic diagram showing how changes since the COVID-19 pandemic are impacting on medical students’ career choices.

Six themes were identified: students’ prior career aspirations, their experience of the medical school curriculum, conceptualisation of general practice, future career predictions, views on the school’s curriculum philosophy, and the influence of the COVID-19 pandemic. The themes interacted with one another, developing sequentially from entry to medical school, experiences and conceptualisation of general practice to development of future career ideas. The curriculum philosophy of each school influenced this journey with the COVID-19 pandemic having a more subtle impact on all themes (see Figure 1).

### Career aspirations on entry to medical school

Students described a variety of thoughts about their career aspirations before starting medical school that were shaped by different experiences; some were clearer than others about their career choice:

‘Before I came into medicine I would see on the telly and you would see like the A&E departments […] you see a lot about A&E and there’s lots of shows […] So I thought oh that’s really exciting […] that’s really cool, really fast paced.’

‘I knew that some of it was hospital and some of it was GP and I knew I didn't want to do the GP stuff. That’s all. I thought it was boring.’

Some participants reported extremely positive experiences of general practice before medical school while others found that it put them off considering general practice as a career or did not tell them enough about the reality of the role.

‘I did like a week in a GP, which now having done it as a medical student, I don’t think was very reflective of the job […] I obviously understand it’s different because you’re not a medical student […] So I didn’t actually get to appreciate much about what being a GP was actually like.’

### Curriculum experiences

Students from all three schools discussed experiences of their general practice placements throughout their curriculum, with both positive and negative outcomes. The quality of organisation and supervision had a large impact on the learning experience, with several students recalling particularly strong placements.

‘I've got so many friends who have just spoken so highly of their practices in third and fourth year because they had really good tutors and those who are willing to supervise them, willing to go the extra mile and do teaching.’

However, positive placement experiences did not always correlate directly with career choice.

‘Throughout medical school I’ve really enjoyed general practice and thought it’s been really good, but I don’t want to be one [a GP].’

Interestingly, students felt they experienced more continuity of care in secondary care placements, when seeing patients from admission to discharge. There were also some aspects of hospital placements that students felt were relevant to general practice.

‘Everyone talks about that longitudinal care and how you really get to know that person. But I have found I got to know patients so much better when I was in the hospital […] because you’re seeing the same person each day and then you sort of get that bit more information about them.’

### Conceptualisation of general practice

Students conceptualised thoughts about general practice work through their clinical placements, discussion with students and clinicians, and observation of their supervisors. Lifestyle and work–life balance were considered in their decision making.

‘I feel like it is it kind of cemented what I thought of general practice. I think they have quite a good work life balance […]’

However, other students observed long working hours in general practice and felt this compared less favourably.

‘I think general practice is actually a lot harder […] But you see GPs that arrive at work at like 07:30/08:00 in the morning and don't leave until 7pm/8pm because just the workload is massive. So I think it’s not actually as, as great as people say.’

Variety, of patients and presentations, was seen as a positive by many students.

‘[…] made me realise the variety in general practice. Which again, like attracted me to it.’

Many students commented that the working environment in general practice was lonely and that hospital placements had greater emphasis on multidisciplinary working and more opportunities to engage with other professionals.

### Predicting the future

Placement experiences helped students to develop career plans, particularly through opportunities to discuss training and career progression. However, it was recognised that placements were not always representative of the end career which limited understanding about the reality of the role.

‘So you’re making high value judgments based off your limited experience of things, but that experience of things isn’t going to be reflective of what you're going to be doing later on in that career.’

A number of students described thoughts about work–life balance and the structure or length of postgraduate training influencing their future career plans. Concerns about working within secondary care for some were identified as a ‘push’ towards general practice.

‘[…] I’ve heard of a lot of people going through medical school and then becoming a hospital doctor and like, burning out and even dropping out of medicine. So I feel like that’s definitely kind of pushing me more towards general practice kind of as opposed to hospital medicine or surgery.’

Students seemed highly aware of the current challenges faced by the NHS and identified that the current way of working within both primary and secondary care may not reflect their future careers.

‘It’s hard because obviously we’re coming into medicine at a time when the NHS is in crisis. So we’re kind of looking at these systems falling apart a little bit and thinking, oh my God, like what’s it going to be like in 10 years.’

### Curriculum philosophy

Students at all three institutions felt general practice was prioritised by their medical school as a future career. Students from all schools reported being told that 50% of them would become GPs, although one student reported hearing the figure was even higher for their institution. Another student, from a different institution, reported being told that their institution produced more GPs than other institutions.

‘That’s what X is known for, isn’t it? We’re a GP factory.’

Students felt an emphasis on general practice ran throughout the curriculum, evidencing early clinical exposure in general practice, use of general practice cases for teaching and many early year tutors being GPs. Teaching of communication and clinical skills were also rooted in general practice.

‘It’s just interesting thinking back to it now and thinking actually, like, so much of our teaching that wasn’t actually at GP practices, was still with a GP head on.’

The perceived large volume of time spent on general practice placements were quoted as evidence of institutions favouring general practice. In contrast, one student stated that they spend so much time in hospital there can be a tendency to forget about general practice, even though they perceived the overall philosophy was to promote general practice as a career.

Students pointed out that positive experiences in any specialty would make a student more likely to consider that specialty as a career, and this was thought to be particularly true for general practice where placement experience was overwhelmingly positive.

‘And I feel like the other placements have been pretty good, but I think this is the one that has the best teaching and the one you have the most hands on as well.’

### COVID-19 pandemic and general practice placements

Unsurprisingly the COVID-19 pandemic interrupted the study and clinical placements of many students, and reduced clinical exposure was linked to a diminished learning experience in general practice.

‘GP placements online sort of hindered my learning quite a bit … so it felt like I didn’t get a good experience till we came into further in phase 2 — have our own clinics and we actually see patients ourselves’.

The pandemic brought changes in patterns of work with more online consultations and remote working. This had a mixed response and highlighted the ‘loneliness’ of the environment for many students.

‘Instinctively didn’t like the idea of doing things virtually, so it would have put me off specialties that were doing more virtual things. But actually, liked doing telephone consultations — probably reversed my opinion.’

‘General practice has probably moved down in the list in the post COVID-19 world because I think they’ve retained a lot more “online stuff” […] doesn’t really appeal to me I want to be with patients in the room all the time. […] comes across as a much more lonely job now because you can be, you know, on the phone doing phone calls all day in your room.’

Students discussed their own perceptions of general practice and those of the public. They also identified the pressure the pandemic had put on health care as a whole.

‘I think COVID-19 kind of changed our experience of general practice and I think public opinion also really changed how GPs were viewed throughout COVID-19 and I think they got a bit of, quite a lot of stick for a lot of the telephone appointments kind of lasting longer in primary care than they did in secondary care and I think that kind of changed opinions’

‘I think that general practice has probably been forever changed by COVID-19.’

‘It’s kind of just exacerbated the workload of an NHS that was already struggling.’

## Discussion

### Summary

Our findings demonstrate the varied inputs that factor into undergraduate students’ decision making about future career choices. Early ideas were formed from several sources including work experience, close family members, and media and television. Many students felt that their medical school placed an emphasis on general practice through several aspects of the curriculum, including the amount of placement time. Positive clinical experiences with high quality supervision in general practice helped students to clarify thoughts about general practice as a career.

Students considered workload and lifestyle when thinking about a career in general practice. Students were aware of current challenges within the NHS, workforce pressures, and the likelihood of further evolution in working patterns, particularly within primary care. The COVID-19 pandemic appears to have exacerbated some of these findings, such as NHS workforce pressures and an increased sense of loneliness.

#### Strengths and limitations

Our study includes a small number of students from three medical schools that may not reflect the range of different placement experiences and curriculum design in UK medical schools. However, certain themes were consistent across all three schools, such as the perception that GP was encouraged as a career choice. Recruitment challenges meant that some focus groups were smaller than intended which may have limited discussion. As a qualitative study from an interpretive paradigm the aim was to generate ideas and theories rather than to seek generalisable findings. The researchers that facilitated the focus groups were all early career GPs, which may have introduced bias into data collection and analysis. Finally, the exact impact of the COVID-19 pandemic on particular stages of the course was challenging to evaluate, as students were comparing early clinical experiences pre-pandemic to later clinical experience during or post-pandemic.

#### Comparisons with existing literature

Denigration of GP as a specialty was not consistently identified as a driver behind career decisions. However, students were aware that they were talking to GP researchers and therefore may not have been willing to reflect negatively on the specialty. Remote consulting has clearly impacted students’ feelings of isolation and loneliness in GP. However, it had also been found before the pandemic that isolation resulting from the nature of general practice can negatively impact the experience of both medical students and GPs.^8,13^

The influence of the COVID-19 pandemic on both clinicians and the system in which they work is new and still evolving, and so further research is required to assess its full impact.

#### Implications for research and practice

The process of making career decisions remains multifactorial and complex, and studies are warranted to explore further some of the current and previous findings. The impact of the pandemic, such as increased remote consulting, in the longer term remains an important consideration as students described varied experiences with both positive and negative outcomes. Some of our novel findings in particular warrant further exploration, such as students’ comments suggesting that continuity of care and the multidisciplinary team are experiences that are more visible in secondary than primary care. Our study supports the continued emphasis on authentic clinical placements^14^ as fundamental to medical education as students noted their lack of clinical exposure during the pandemic.

Our study has confirmed previous findings that clinical experiences, the perceived narrative of the school, work–life balance and working environment remain important to students in making career plans. However, in addition, we have found the changing landscape in general practice since the COVID-19 pandemic, including remote consulting, workload, continuity of care and team-working, are additional factors that concern students.

The magnitude of the impact of the COVID-19 pandemic remains unclear, although it appears to have had a negative impact on students’ perceptions of working in the NHS and in general practice. When making career choices, students are clearly aware of the current challenges within UK healthcare and are apprehensive about how this may affect their working lives.

## Data

The dataset relied on in this article is available from the corresponding author on reasonable request.
